# Untangling the Influences of Voluntary Running, Environmental Complexity, Social Housing and Stress on Adult Hippocampal Neurogenesis

**DOI:** 10.1371/journal.pone.0086237

**Published:** 2014-01-23

**Authors:** Catherine-Alexandra Grégoire, David Bonenfant, Adalie Le Nguyen, Anne Aumont, Karl J. L. Fernandes

**Affiliations:** Department of Pathology and Cell Biology, Groupe de recherche sur le système nerveux central (GRSNC), and Center of Excellence in Neuroscience of the Université de Montréal (CENUM), Université de Montréal, Montréal, Canada; University of Victoria, Canada

## Abstract

Environmental enrichment (EE) exerts powerful effects on brain physiology, and is widely used as an experimental and therapeutic tool. Typical EE paradigms are multifactorial, incorporating elements of physical exercise, environmental complexity, social interactions and stress, however the specific contributions of these variables have not been separable using conventional housing paradigms. Here, we evaluated the impacts of these individual variables on adult hippocampal neurogenesis by using a novel “Alternating EE” paradigm. For 4 weeks, adult male CD1 mice were alternated daily between two enriched environments; by comparing groups that differed in one of their two environments, the individual and combinatorial effects of EE variables could be resolved. The Alternating EE paradigm revealed that (1) voluntary running for 3 days/week was sufficient to increase both mitotic and post-mitotic stages of hippocampal neurogenesis, confirming the central importance of exercise; (2) a complex environment (comprised of both social interactions and rotated inanimate objects) had no effect on neurogenesis itself, but enhanced depolarization-induced c-Fos expression (attributable to social interactions) and buffered stress-induced plasma corticosterone levels (attributable to inanimate objects); and (3) neither social isolation, group housing, nor chronically increased levels of plasma corticosterone had a prolonged impact on neurogenesis. Mouse strain, handling and type of running apparatus were tested and excluded as potential confounding factors. These findings provide valuable insights into the relative effects of key EE variables on adult neurogenesis, and this “Alternating EE” paradigm represents a useful tool for exploring the contributions of individual EE variables to mechanisms of neural plasticity.

## Introduction

It has been known for over half a century that the surrounding environment affects the anatomy, chemistry, and functional properties of the brain [1]–[4]. Early studies showed that rodents exposed to environmental enrichment (EE), consisting of large cages, social enrichment and diverse multisensory stimulation, displayed increased brain sizes, altered neurotransmitter levels and behavioral changes [3], [5]–[7]. Likewise, in humans, life experiences such as spatial memory training [8]–[10], cardiovascular exercise [8], [11], [12], sensory deprivation [9] and stress [10], [13] can affect learning and memory. The profound effect of environmental parameters on brain function has led to the widespread use of EE paradigms as tools for both research and rehabilitation. However, our basic understanding of this phenomenon remains remarkably nebulous. Diverse EE paradigms are now used, with little understanding of how differences in individual EE variables (such as physical exercise, cognitive stimulation, stress, and social interactions) might impact on specific downstream biological mechanisms, such as changes in neurotrophic factor synthesis [14], dendritic growth [15]–[17], synaptic plasticity [18], electrophysiological properties [19] and adult neurogenesis [20].

The hippocampal dentate gyrus (DG) is a rare niche where neurogenesis is preserved throughout life [21]–[24]. DG neurogenesis is a multi-step process in which radial glia-like precursors generate proliferating progenitors and neuroblasts that mature into DG granule neurons implicated in learning, memory and mood regulation [25]–[29]. It is now well established that EE modulates adult hippocampal neurogenesis [20], [30]. Previous studies have identified physical activity as an important proneurogenic stimulus within EE [31]–[33]. However, previously used housing paradigms could not unambiguously separate the effects of running from other EE variables, such as environmental complexity, social context and stress, which are also reported to influence neurogenesis [34]–[38]. Clearly defining the relative and/or combinatorial effects of such variables is essential for the rational design of EE paradigms, as EE can improve some cognitive functions at the expense of others [8], and can have unexpected consequences under pathological conditions [39], [40].

Here, we developed a novel “Alternating EE” paradigm to experimentally isolate EE variables and to gain insights into their specific contributions to adult hippocampal neurogenesis.

## Materials and Methods

A total of 129 two-month-old male CD1 mice (Charles River, Senneville, QC, Canada) and eight 2-month-old male C57BL/6 mice (Charles River, Senneville, QC, Canada) were used in these studies. All experiments were conducted in accordance with the guidelines of the Canadian Council of Animal Care and were approved by the Animal Care committee of the Université de Montréal.

### Housing conditions and Experimental groups: Alternating EE paradigm

**Figure 1:**
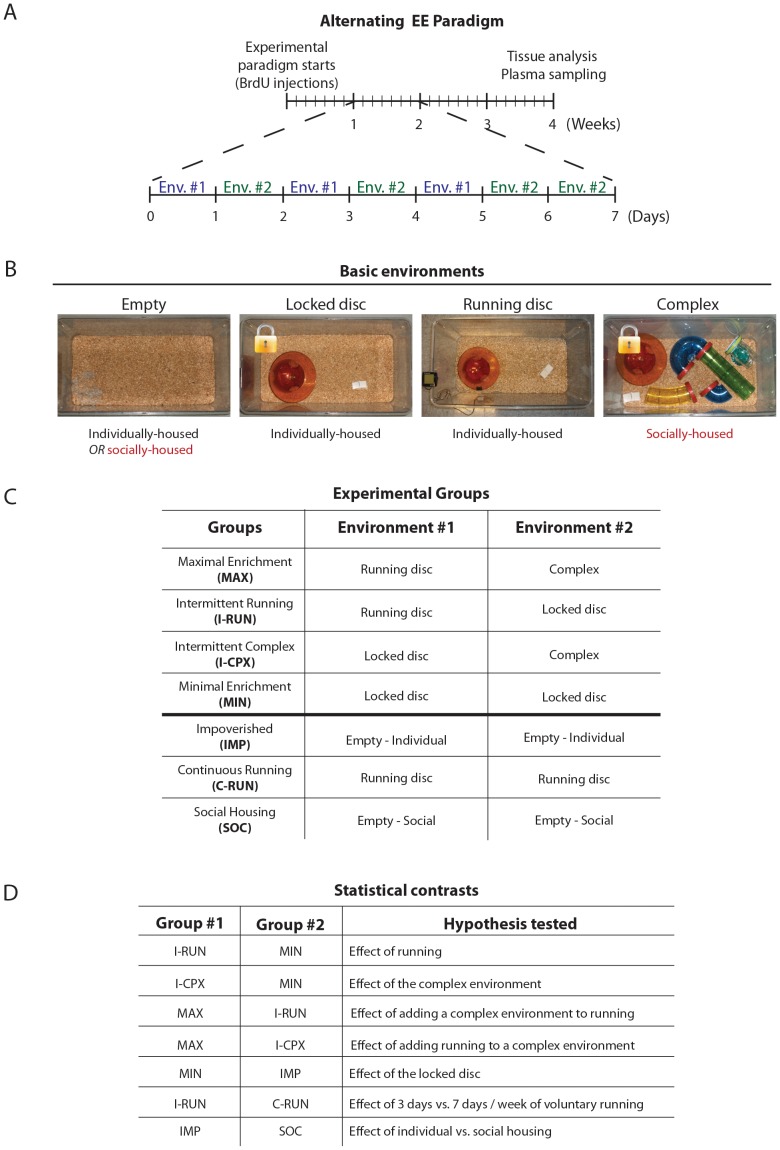
The Alternating EE paradigm. **A**, Timeline of the Alternating EE paradigm. Each experimental group was alternated between two types of housing environments 6 times/week for a period of 4 weeks. Each mouse received two injections of BrdU on the first day of the paradigm. **B**, Basic environments used in the Alternating EE paradigm: *Empty* (devoid of all items, in which mice are either isolated or socially housed in groups of 3), *Locked disc* (containing a running disc that has been locked to prevent running exercise), *Running disc* (containing a normal running disc), and *Complex* (a *Locked disc* cage to which multi-colored tunnels have been added (rotated 4 times/week) and 3 mice are socially housed) (see Methods for additional details). **C**. Alternating EE experimental groups: Each experimental group was built using 2 of the basic environments in B. Mice in each group alternated daily between these two basic environments for the entire 4 week period. **D**, Statistical contrasts: For each parameter measured in this study, a difference detected by one-way ANOVA was followed by the testing of 7 specific and pre-determined hypotheses. Each of these 7 statistical contrasts is made between two experimental groups that differ in only one EE variable. No statistical analyses were made between groups in which more than one variable differed.

All animals were provided with food and water *ad libitum*, and all environments contained nesting material and a basic litter (PRO-CHIP 8–16, PWI brand). Four types of housing environments were used in the Alternating EE paradigm, as shown in Fig. 1B and detailed here:

#### Empty environment

Animals were housed in empty 24.0 cm×44.0 cm×20.0 cm rat cages. Mice were housed either individually or in groups of 3, depending on whether they were in the Impoverished or Social housing experimental groups (below).

#### Locked disc environment

Animals were housed in 24.0 cm×44.0 cm×20.0 cm rat cages containing a locked or unlocked running disc (Red mouse igloo, K3327, and amber fast-trac running disc, 7.5 cm in diameter, K3250, Bio-Serv, Frenchtown, NJ, USA). Running cages were outfitted with odometers (Sigma BC509) to measure the running distance. Mice were housed individually.

#### Running disc environment

Identical to the Locked disc environment except that the running disc was unlocked to permit voluntary running.

#### Complex environment

Animals were housed in 24.0 cm×44.0 cm×20.0 cm rat cages that contained an igloo, locked running disc and colored tunnels for hamsters (Habitrail, 8 inches green trail, yellow curve, blue U-turn, transparent tee and blue elbow). Tunnels were re-oriented and their conformations re-arranged at each cage alternation (4 times/week). Mice were housed in groups of 3. This *Complex* environment thus provides social interactions, inanimate objects, and frequent conformational novelty.

#### Experimental groups used in the Alternating EE paradigm

The Alternating EE paradigm was repeated using two separate cohorts of mice (n = 36–42/cohort) in order to obtain sufficient tissues for analysis of all markers. One cohort received two intraperitoneal injections of 5-bromo-2-deoxyuridine (BrdU, Sigma-Aldrich, Oakville, ON, Canada, 100 mg/kg) at 9am and 4pm on the first experimental day to assess cell survival. Mice were randomized and separated into one of 7 different groups. Each group alternated between 2 housing conditions, 6 times per week for 4 weeks, as shown in Fig. 1A–D and described here: 1) Maximal enrichment (“MAX”, n = 6) mice were alternated between the *Running disc* and the *Complex* environments, 2) Intermittent Running (“I-RUN”, n = 6) mice were alternated between the *Running disc* and the *Locked disc* environments, 3) Intermittent Complex Environment (“I-CPX”, n = 6) mice were alternated between the *Locked disc* and the *Complex* environments, 4) Minimal enrichment (“MIN”, n = 6) mice were alternated between identical *Locked disc* environments, 5) Impoverished (“IMP”, n = 6) mice were alternated between identical *Empty* environments and were housed individually, 6) Continuous Running (“C-RUN”, n = 6) mice were alternated between identical *Running disc* environments, 7) Social housing (“SOC”, n = 6) mice were alternated between identical *Empty* environments and were housed in groups of 3. Mice used for analysis of c-fos immediate early gene expression remained within their enriched environments until time of anaesthesetic overdose.

### Housing conditions: Strain comparison, Handling effect, and Wheel versus Disc paradigms

All animals were provided with food and water *ad libitum*, and all environments contained nesting material and a basic litter (PRO-CHIP 8–16, PWI brand).

#### Strain Comparison

2-month-old adult male C57BL/6 mice (n = 8) and 2-month-old adult male CD1 mice (n = 8) were individually housed in 24.0 cm×44.0 cm×20.0 cm rat cages. Mice were exposed to either *Empty* (n = 4/strain) or *Running disc* environments (n = 4/strain) for 4 weeks.

#### Handling Effect

2-month-old adult male CD1 mice (n = 12) were individually housed in 24.0 cm×44.0 cm×20.0 cm rat cages. Mice were separated into No Handling and Handling groups (n = 6/group). The No Handling group was exposed to the *Empty* environment for the entire period and was only handled only twice (cage changing), while the Handling group was treated as for the Intermittent EE paradigm, i.e., holding the mouse by the tail and transferring it to another cage 6 days a week for a total of 24 times during the 4-week experimental period.

#### Wheel vs. Disc

2-month-old adult male CD1 mice (n = 25) were randomized and separated into one of the five environments (n = 5/group): 1) *Empty*, 2) *Locked wheel*, 3) *Locked disc* 4) *Running wheel* and 5) *Running disc*. Mice were individually housed in either 17.0 cm×28.0 cm×12.5 cm mouse cages (*Empty*), 12.7 cm×20.3 cm×35.6 cm cages (*Locked* and *running wheel*, 22.9 cm diameter wheel) or 24.0 cm×44.0 cm×20.0 cm rat cages (*Locked* and *running disc*).

### Tissue Preparation

Mice received a lethal dose of chloral hydrate (7%), followed by a dose of Xylazine (0.1%) and were then perfused trans-cardially with 30 mL of 1× phosphate-buffered saline (PBS, pH 7.4) (PBS 10×, Wisent, 311-012-CL), followed by 40 mL of 4% formaldehyde fixative solution (freshly hydrolyzed from 4% paraformaldehyde, pH 7.4, Fisher, T353-500). The brains were removed and post-fixed in 4% formaldehyde overnight and then kept in PBS at 4°C until sectioning. The entire brain of each animal was cut into 40 µm coronal sections using a vibrating microtome (Leica VT1000S, Leica Microsystems, Richmond Hill, ON, Canada), and the tissue sections were stored at −20°C in an antifreeze solution (glycerol∶ethylene glycol∶PBS 1×, 3∶3∶4).

### Immunohistochemistry

Primary antibodies used in this study were mouse anti-human Ki67 (1∶200, BD Biosciences, Mississauga, ON, Canada, 556003), goat anti-rabbit NeuroD (1∶500, Santa Cruz Biotechnology, Santa Cruz, CA, SC-1084), goat anti-human Doublecortin (DCX; 1∶500, Santa Cruz Biotechnology, SC-8066), rabbit anti-human Calretinin (1∶2500, Swant, Bellinzona, Switzerland, CR7699/3H), rabbit anti-human c-fos (1∶5000 for fluorescence, 1∶20000 for DAB, Calbiochem, San Diego, CA, PC38), mouse anti-cow S100β (1∶1000, Sigma-Aldrich, Oakville, ON, Canada, S2532), rat anti-BrdU (1∶800, AbD Serotec, Oxford, UK, MCA2060) and mouse anti-mouse Neuronal nuclei (1∶100, NeuN, Milllipore, Temecula, CA, MAB377).

For Calretinin and c-fos immunohistochemistry and BrdU/NeuN/c-fos triple immunofluorescence, the labeling procedure was performed as previously described [35]. For Ki67 and NeuroD immunohistochemistry, the protocol was modified to include an antigen retrieval step. Free-floating 40-µm sections were washed in PBS, mounted onto glass slides, post-fixed with 4% formaldehyde solution for 10 minutes, washed with PBS, and then incubated for 40 minutes in a Citrate-EDTA (10 mM Citric Acid, 2 mM EDTA, 0.05% Tween 20, pH 6.2) antigen retrieval solution. They were then blocked for 2 hours in 4% bovine serum albumin (BSA)/0.1% Triton-X/PBS (for Ki67) or 10% normal donkey serum (NDS)/0.1% Triton-X/PBS (for NeuroD). Sections were incubated overnight at room temperature in primary antibodies diluted in either 2% BSA or 5% NDS in PBS.

### Corticosterone assay

Blood samples were collected from the anaesthetized animals, prior to cardiac perfusion, using a 23 gauge needle inserted into the posterior *vena cava*. Interval between anaethesia injection and blood collection was approximately 5 minutes. Blood samples were transferred to a microtainer containing K_2_EDTA (BD Biosciences, Mississauga, ON, 365974), inverted 20 times, and the plasma extracted after centrifuging at 1000 g for 15 minutes at 4°C. Samples were stored at −80°C. Plasma was diluted 1∶200 and plasma corticosterone concentrations were assayed in duplicate using an enzyme-linked immunoassay kit, according to the manufacturer instructions (Cayman Chemical, Ann Arbor, MI; #500655).

### Cell quantifications

For the Alternating EE paradigm, the number of SGZ/GZ cells positive for Ki67, NeuroD, Calretinin, and BrdU was quantified on every 6th section between Bregma −1.06 mm and −2.98 mm of the hippocampus (8 sections total/marker/animal). The raw cell counts were corrected for oversampling due to split cells by multiplying by (1 - object diameter/section thickness), where the object diameter refers to the average diameter of the marker in question. Mean object diameters were determining by measuring the diameter of 100 positive cells for each marker (NIH ImageJ, 64-bit Java software for Mac), and yielded correction factors of 0.77 (Ki67, NeuroD), 0.75 (Calretinin) and 0.79 (BrdU, c-fos). The corresponding SGZ/GZ reference volumes of the sections were determined using the Cavalieri principle (grid size of 10 microns, 20× objective) in StereoInvestigator (MBF Bioscience, VT). The mean cell density was then obtained by dividing the corrected total number of marker-positive cells on the sampled sections by the sum of the section SGZ/GZ reference volumes. Results are expressed as density of marker-positive cells per mm^3^ of SGZ/GZ. Cell counts were performed manually by a blinded observer using a 40× objective, and slide codes were only broken after all quantifications had been completed for any given marker.

For the control experiments (Strain comparison, Handling, Wheel vs. Disc), raw cell counts were corrected for oversampling as above and then multiplied by 6 to obtain an estimate of the total number of marker-positive cells between the Bregma coordinates.

Triple immunofluorescence stainings for BrdU/NeuN/c-fos and for BrdU/S100beta/DCX were performed to determine i) the proportion of c-fos-positive cells that co-express the mature neuronal marker NeuN, and ii) the proportions of BrdU-positive cells that co-express NeuN, DCX or S100beta. To do so, each c-fos or BrdU-positive cell in a complete 1-in-6 series of sections was brought into focus in turn using a 40× objective (400× total magnification) and scored for absence or presence of coexpression of the co-labels. In the case of BrdU/NeuN/c-fos staining, the NeuN antibody penetration through the tissue was observed to be incomplete; to avoid obtaining false NeuN-negative cells, NeuN double-labelling analyses were therefore restricted to the z-levels of NeuN antibody penetration.

### Statistical analyses

For the Alternating EE paradigm, statistical analyses were performed using SAS 9.3 statistical analysis software (SAS Institute). All experimental groups were first analysed together by One-way ANOVA. Rejection of the null hypothesis was followed by the application of specific contrasts (linear combination of the means) that tested 7 pre-defined hypotheses summarized in Fig. 1D. Only these 7 specific comparisons between experimental groups were made, in order to restrict statistical analyses to groups that differ in only one experimental variable. Significance level was set at α = 0.05. Error bars represent standard error of the mean.

For the control experiments (Strain comparison, Handling and Wheel vs Disc), statistical comparisons between groups were performed using t-tests (Graph-Pad Prism, Windows version 5.0a). Significance level was set at α = 0.05. Error bars represent standard error of the mean.

## Results

### Design of the Alternating EE paradigm

To effectively isolate the effects of running, environmental complexity, social interactions and stress, we devised an “Alternating EE” paradigm that would: 1) allow for the stepwise addition/subtraction of EE variables between conditions of maximal and minimal enrichment, 2) preserve as much uniformity as possible across groups, enabling statistical comparisons to be focused on groups for which only a single independent variable had been altered, and 3) in the case of running environments, permit individualized measurement of running distances. The Alternating EE paradigm is based on intermittent exposures to EE (Fig. 1A). Over a 4-week period, adult male mice were alternated daily between two types of basic environments (6 times per week); by comparing experimental groups in which only one of the two environments differed, individual EE variables could be effectively isolated.

Four basic environment types were used to construct the Alternating EE experimental groups (detailed in the Methods and shown in Fig. 1B): an *Empty* environment that was devoid of all objects (1 mouse or 3 mice per cage); a *Locked disc* environment containing a non-functional running disc apparatus (1 mouse per cage); a *Running disc* environment providing voluntary access to a functional running disc (1 mouse per cage); and a *Complex* environment that consisted of inanimate objects (a locked disc and colored tunnels), social enrichment (3 mice per cage), and conformational novelty (change of object organization).

Four Alternating EE experimental groups (Fig. 1C) were used to test for primary influences of the *Running disc* and the *Complex* environments. The Maximal Enrichment (“MAX”) group was sequentially exposed to both the *Running disc* and the *Complex* environments. The Intermittent Running (“I-RUN”) and Intermittent Complex (“I-CPX”) groups alternated between a *Locked disc* environment and either a *Running disc* or *Complex* environment, respectively. A Minimal Enrichment (“MIN”) group served as the baseline control group and alternated between identical *Locked disc* environments. This approach allows statistical comparisons to be focused on groups that spend about half their time in identical environments, and enabled us to test for individual and combinatorial effects of the running and complex environment variables. Besides these four main Alternating EE groups, three additional groups were also used (Fig. 1C): an Impoverished (“IMP”) group that alternated between *Empty* environments and was individually housed (allowing assessment of the effect of the Locked disc by comparison to the MIN group), a Continuous Running (“C-RUN”) group that alternated between *Running disc* environments (allowing assessment of differences between 3 and 7 days of running by comparison to the I-RUN group), and a Social Housing (“SOC”) group that alternated between *Empty* environments but that was housed 3 mice/cage (allowing assessment of differences between individual and social housing by comparison to the IMP group). A summary of the 7 pre-determined groupwise comparisons and hypotheses is presented in Fig. 1D.

Several additional features of the experimental design should be noted. First, we used a 4-week experimental paradigm in order to focus on the effects of longer term EE, as previous studies have shown transient effects of some forms of EE on certain aspects of neurogenesis [41], [42]. Second, animals were individually housed during their exposure to running discs, ensuring that running data could be collected for each animal. Lastly, we used outbred CD1 mice rather than the more commonly used C57BL/6 strain, in order to ensure that weaker neurogenic effects of EE are not masked by the high baseline neurogenesis reported in C57BL/6 mice [43]. Indeed, in our own control experiments, we found that male CD1 mice exhibited a 47% lower baseline proliferation rate compared to age-matched C57BL/6 mice (C57BL/6: 2672±166.9 vs. CD1: 1427±129.2 Ki67^+^ cells/DG), permitting a running-induced increase to be detectable with as little as 4 mice/group in CD1 but not C57BL/6 mice (C57BL/6 RUN: 2696±281.8 vs. CD1 RUN: 2639±240.2 Ki67^+^ cells/DG) (not shown).

### Running, but not the *Complex* environment, stimulates hippocampal neurogenesis

**Figure 2:**
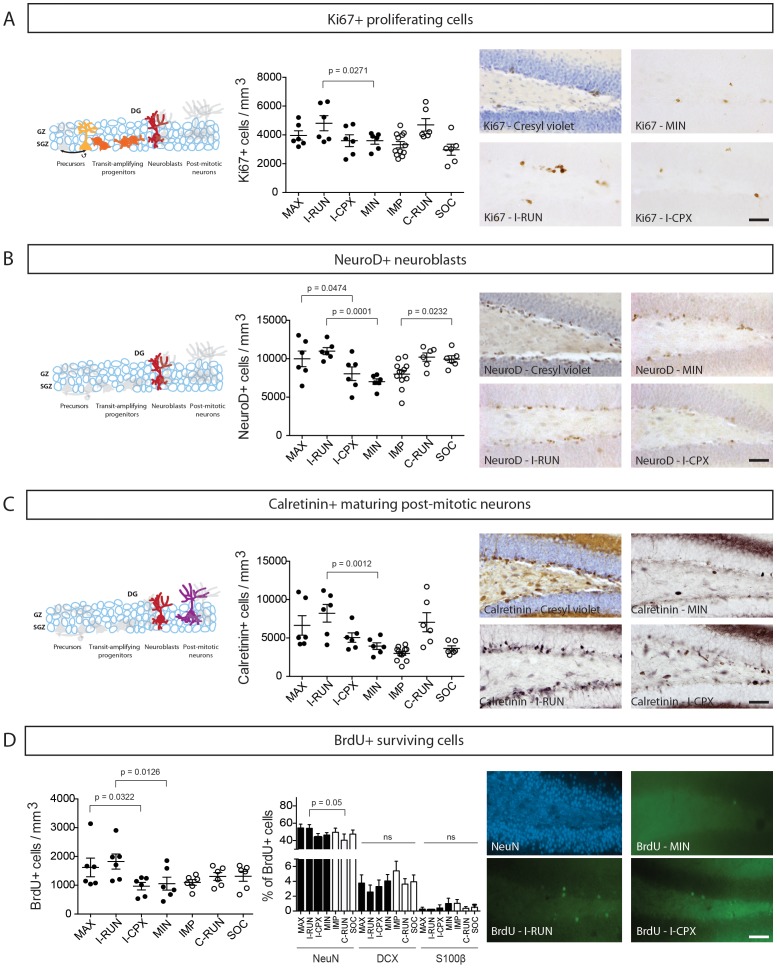
Effects of Alternating EE on the main stages of dentate gyrus neurogenesis. **A–D**, Quantification of the density of **A**, Ki67^+^ proliferating cells, **B**, NeuroD^+^ neuroblasts, **C**, Calretinin^+^ maturing post-mitotic neurons, and **D**, BrdU^+^ surviving cells. At the right of each panel is a sample cresyl-violet counterstaining (A–C) or NeuN-labeling (D) (upper left image), and representative non-counterstained sections from MIN (upper right), I-RUN (lower left) and I-CPX (lower right) experimental groups. Note that a main significant effect of the *Running disc* environment (I-RUN) was detected for all four neurogenesis markers, while the *Complex* environment (I-CPX) did not have a main effect on any marker and did not potentiate the effects of running. In D, middle panel, quantification of the percentage of BrdU+ cells that co-express NeuN, DCX or S100β. See Results for further details. Scale bar = 50 µm. DG = Dentate Gyri.

We began by examining whether the Alternating EE groups exhibit differences in key parameters of adult neurogenesis (proliferation, neuroblast, immature neuron, and survival). Immunohistochemistry was performed for Ki67^+^ proliferating cells (Fig. 2A), NeuroD^+^ neuroblasts (Fig. 2B), Calretinin^+^ maturing post-mitotic neurons [44], [45] (Fig. 2C), and BrdU+ surviving cells (Fig. 2D). Since one-way ANOVA showed differences for all four markers, statistical contrasts were applied to test our 7 pre-determined hypotheses. As shown in Fig. 2A–D, running had a significant effect on all four neurogenic markers compared to the locked disc (I-RUN vs. MIN groups). Conversely, the Complex environment had no significant effect compared to the locked disc (I-CPX vs. MIN). While running increased the density of NeuroD+ and BrdU+ cells when combined with a complex environment (MAX vs. I-CPX), complex environment failed to potentiate the effects of Running at any stage of neurogenesis (MAX vs. I-RUN). Since the locked disc itself did not have an elevated baseline neurogenesis (MIN vs. IMP), it is not responsible for masking possible neurogenic effects of the Complex environment. Besides running, the only variable impacting neurogenesis was social housing: socially housed mice in the impoverished environment exhibited a small but significant increase in NeuroD+ neuroblasts compared to individually housed impoverished mice (IMP vs. SOC). Co-labelling of the surviving BrdU+ cells showed that there was no primary effect of running or the complex environment on the proportions of BrdU+ cells that had acquired phenotypes of NeuN+ mature neurons, DCX+ neuroblasts or S100β+ astrocytes. I-RUN mice had a small but significant increase in NeuN-labelled BrdU+ cells compared to C-RUN mice.

Interestingly, Ki67, NeuroD, Calretinin and BrdU+ cells were all increased to a similar extent in mice that ran 3 d/week versus 7 d/week (I-RUN vs C-RUN groups). In fact, comparison of the three groups containing running mice (MAX, I-RUN and C-RUN groups) showed that despite the fact that C-RUN mice ran 2–3× greater total distances on average (MAX = 130.5±16.92 km; I-RUN = 153.8±15.09 km; C-RUN = 329.5±27.26 km), they did not achieve higher levels of any neurogenic marker than intermittently running I-RUN or MAX mice (Fig. 2A–D).

These results demonstrate that intermittent exposure to running discs for 4 weeks significantly increases the proliferative, neuroblast, post-mitotic and cell survival stages of DG neurogenesis, while comparable exposure to a complex environment comprised of inanimate objects, social interactions and conformational novelty has no effects on these stages of neurogenesis.

### The *Complex* environment, but not running, increases depolarization-associated c-fos expression

**Figure 3:**
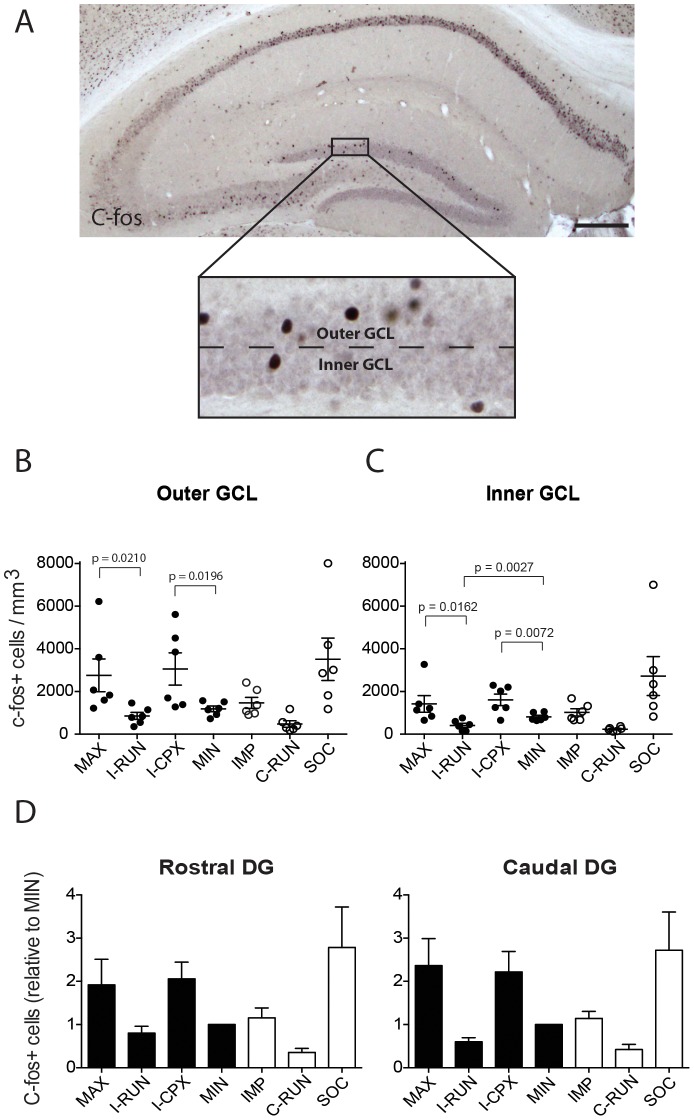
Effects of Alternating EE on depolarization-associated c-fos expression. **A**, Low magnification image of c-fos immunohistochemistry on a coronal section of the hippocampus. Dotted line in the enlarged box illustrates how the granule cell layer (GCL) was divided into outer GCL and inner GCL for quantification purposes. **B,C** Quantifications of c-fos expression in the **B**, Outer GCL and **C**, Inner GCL. Note that a positive effect of the *Complex* environment (I-CPX) was detectable in both the Inner GCL and Outer GCL, while the *Running disc* environment (I-RUN) had a negative effect in the Inner GCL only. Socially-housed mice in empty cages (SOC) also exhibited increased c-fos expression compared to isolated mice (IMP). See Results for further details. **D**, Rostral-caudal distribution of c-fos-expressing cells in the DG. The rostral and caudal tissue sections correspond to the dorsal and ventral hippocampus regions, respectively. Note that the pattern of c-fos expression across groups is virtually identical in both the rostral and caudal DG. Scale bar: 250 µm. DG = Dentate Gyri. GCL = Granule Cell Layer.

To determine whether the Alternating EE groups exhibited differences in the pattern of DG activation, we analyzed the numbers of dentate granule cells expressing the depolarization-induced immediate early gene, c-fos (Fig. 3). Since dentate granule neurons are generated in an outside-in layering pattern during development, with older DG neurons being found in the outer granule cell layer and more recently born neurons in the inner region [46], we quantified inner and outer c-fos-expressing DG neurons separately (Fig. 3A). In contrast to its lack of effects on neurogenesis, the complex environment significantly increased the density of c-fos-expressing cells in both the inner and outer GCL compared to the locked disc (I-CPX vs. MIN)(Fig. 3B,C). Conversely, running did not alter c-fos expression in the outer GCL and reduced its expression in the inner GCL (I-RUN vs. MIN)(Fig. 3B,C). Running did not modify the ability of the complex environment to increase c-fos expression (MAX vs. I-CPX), while the complex environment increased c-fos expression in intermittently running mice (MAX vs. I-RUN). Mice running for 3 or 7 days per week exhibited low levels of c-fos expression (I-RUN vs. C-RUN), with C-RUN mice tending towards lower levels (p = 0.0967 in outer GCL, p = 0.1044 in inner GCL). The locked disc had no difference in c-fos compared to isolated impoverished mice (MIN vs. IMP). Interestingly, social housing tended to increase c-fos expression compared to isolated mice (IMP vs. SOC) (p = 0.0548 in outer GCL, p = 0.0754 in inner GCL), suggesting that the social interaction component of a complex environment is responsible for its stimulation of c-fos expression.

**Figure 4:**
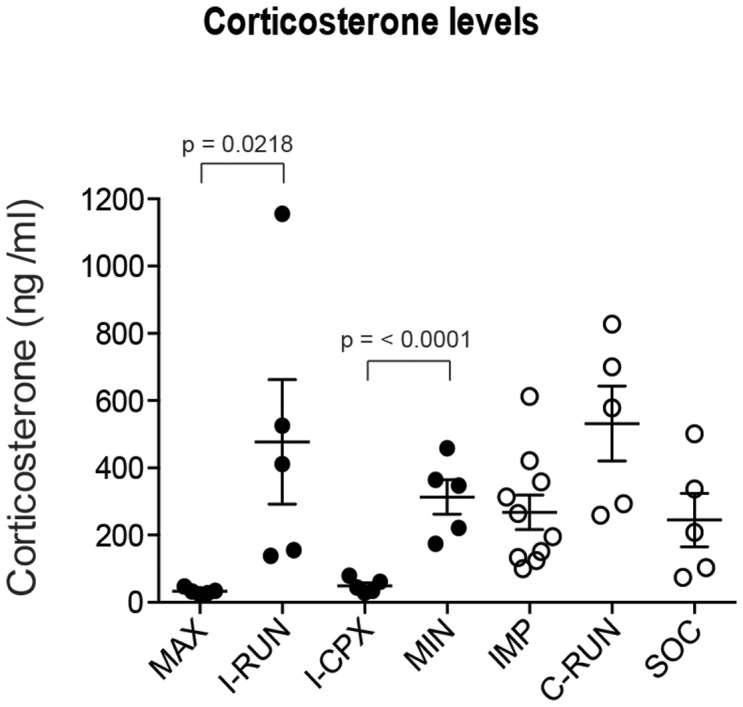
Plasma corticosterone concentrations are reduced in the Complex environment. Plasma corticosterone concentration was measured by ELISA. Note that corticosterone levels are high in all experimental groups except those that include the Complex environment (I-CPX, MAX) See Results for further details.

Since the dorsal and ventral DG play roles in distinct hippocampal-dependent functions [47], [48], we also separated the c-fos quantifications according to rostral (dorsal) and caudal (ventral) sections and found that the effects of the *Complex* environment on c-fos expression were not regionally specific: c-fos-expressing cells were distributed across both rostral and caudal dentate gyrus, and the *Complex* environment stimulated c-fos expression to a similar extent in both regions (Fig. 4D).

These results indicate that the complex environment increases depolarization-associated c-fos expression in the DG, likely mediated by the social interaction component, while running decreases basal c-fos expression.

### The *Complex* environment reduces plasma corticosterone

Stress has been shown to negatively regulate hippocampal neurogenesis [49], [50], but paradoxically, increased levels of circulating stress hormones have also been positively associated with EE-induced neurogenesis [42], [51]. In order to test whether chronic changes in stress-induced hormones are observed and potentially involved in the persistent effects of the *Running disc* and/or the lack of effects of *Complex* environment on adult neurogenesis, we assessed blood plasma levels of the stress-induced hormone, corticosterone, at the end of the 4-week Alternating EE paradigm (Fig. 4).

Compared to the locked disc environment, plasma corticosterone concentrations were diminished by the complex environment (I-CPX vs. MIN) and unchanged by the running environment (I-RUN vs. MIN). There was no difference in corticosterone concentrations in mice running for 3 or 7 days per week (I-RUN vs. C-RUN). The suppressive effect of the complex environment still occurred when used in alternation with running (MAX vs. I-RUN), while running did not alter the effect of the complex environment (MAX vs. I-CPX). Corticosterone levels were not affected by the presence of the locked disc (MIN vs. IMP) or by individual vs. social housing of mice in the impoverished environment (IMP vs. SOC).

These data yield several informative observations. First, since groups exposed to the complex environment (I-CE and MAX) have low plasma corticosterone, their lack of neurogenic effects cannot be attributed to elevated stress. Second, since running groups with both high corticosterone (I-RUN and C-RUN) and low corticosterone (MAX) displayed comparable running-induced increases in neurogenesis (Fig. 2), chronically increased corticosterone is neither required for nor adversely affects running-induced neurogenesis. Third, since two of the elements in the complex environment (social interactions and the locked disc) had no effect on corticosterone levels by themselves, it is likely that the regularly rotated tunnels are essential for the corticosterone suppression observed.

### Absence of neurogenic effects of the *Complex* environment is not due to daily handling or type of running apparatus

Our preceding data using the Alternating EE paradigm indicated that the *Complex* environment enhances DG neuronal activity but does *not* enhance adult neurogenesis. We next sought to eliminate the possibility that neurogenic effects of the *Complex* environment might be masked by intrinsic features of our Alternating EE paradigm: specifically, by an increased basal level of neurogenesis due to i) the daily handling of mice associated with alternation between environments or ii) the use of horizontal running discs versus vertical running wheels.

**Figure 5:**
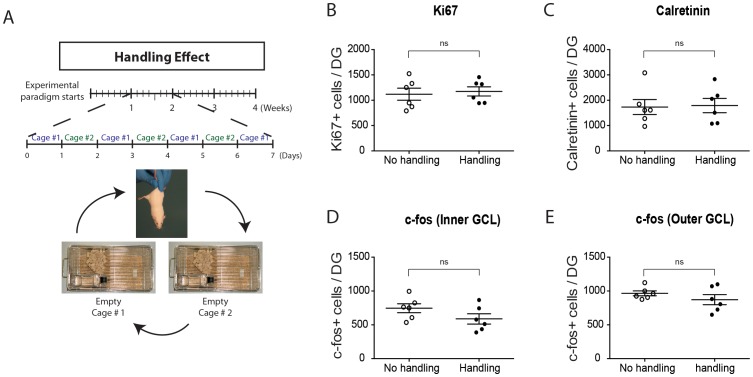
Daily handling does not affect basal neurogenesis in the Alternating EE paradigm. **A**, Timeline of the Handling Effect experiment. Mice were either housed as for the IMP group of the Intermittent EE paradigm (i.e., *Empty* environment with daily handling) or in an *Empty* housing condition without daily handling for 4 weeks. **B–E**, Quantifications of the number of **B**, Ki67^+^ proliferating cells, **C**, Calretinin^+^ maturing post-mitotic neurons, **D**, Inner GCL c-fos^+^ cells or **E**, outer GCL c-fos^+^ cells. No significant effects of daily handling on these markers were detected (t-tests). See Results for statistical details. DG = Dentate Gyri.

To evaluate whether four weeks of daily handling was capable of altering the basal levels of hippocampal neurogenesis (Fig. 5), we repeated the handling that was executed between two *Empty* cages (i.e., IMP group) during the main experimental paradigm and compared the results with a group of mice that was maintained for 4 weeks without any daily handling (Fig. 5A). The Handling group was handled 6 times per week while the No Handling group was only manipulated only once every 2 weeks (cage changing). Quantification of immunohistochemical results showed no changes in the estimated numbers of Ki67^+^ cells (Fig. 5B), Calretinin^+^ cells (Fig. 5C), and c-fos+ cells (Fig. 5D,E). These data indicate that the absence of effects of the *Complex* environment on neurogenesis is not due to a handling-induced increase in basal neurogenesis.

**Figure 6:**
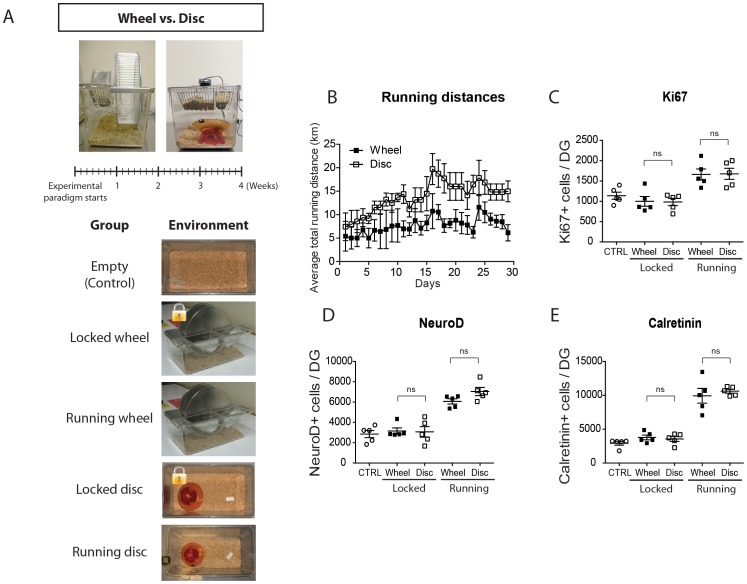
Comparison of the effects of running wheels and running discs on adult neurogenesis. **A**, The Wheel versus Disc experiment. Mice were maintained for 4 weeks in one of five housing conditions: Empty, Locked wheel, Running wheel, Locked disc and Running disc. **B**, Comparison of average daily running distances on Running Wheels and Running Discs. Note that average running distances were about 80% higher on Discs than on Wheels. **C–E**, Quantifications of the numbers of **C**, Ki67^+^ proliferating cells, **D**, NeuroD^+^ neuroblasts and **E**, Calretinin^+^ maturing post-mitotic neurons. In all cases, there was no significant difference between the Control, Locked wheel and Locked disc groups, or between the Running wheel and Running disc groups (t-tests). DG = Dentate Gyri.

Since several types of running equipment are used in the EE paradigms in the literature, we also tested the possibility that the horizontal running discs and igloos used in the present study might serve as greater baseline enrichment than commonly used vertical running wheels [33], [41], [52], [53]. We therefore compared neurogenesis in mice following 4 weeks of exposure to an Empty cage, to Locked or Running wheels, or to the Locked or Running discs used throughout this study (Fig. 6A). Interestingly, mice running on discs ran 84% more than mice on running wheels (Running disc: 13.80 km/day, Running wheel: 7.49 km/day; p<0.0001, Fig. 6B). Immunohistochemical analysis and quantification revealed that mice exposed to Locked wheels and Locked discs had equal estimated numbers of Ki67^+^ cells (Fig. 6C), NeuroD^+^ cells (Fig. 6D), and Calretinin^+^ cells (Fig. 6E), and no difference compared to the control mice, confirming that the locked running apparatus did not have any persistent effects on neurogenesis. Mice housed with Running wheels or Running discs likewise achieved equal levels of running-induced Ki67^+^ cells, NeuroD^+^ cells and Calretinin^+^ cells (Fig. 6C–E), despite their significantly greater running distances on discs than on wheels.

These findings reveal that the absence of neurogenic effects of the *Complex* environment in the Alternating EE paradigm is not due to masking by an elevated basal rate of neurogenesis caused by the mouse handling or the type of running apparatus.

## Discussion

**Figure 7:**
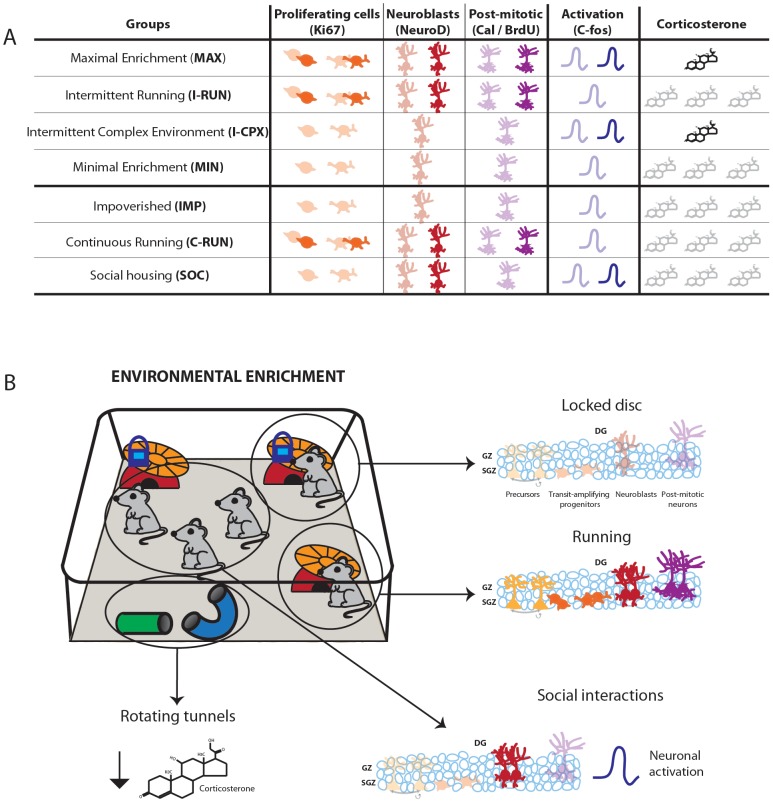
Summary of long-term effects of individual EE variables on hippocampal neurogenesis, c-fos expression and corticosterone levels. **A**, Table summarizing relative changes to DG neurogenesis, depolarization-associated c-fos expression within the granule cell layer, and plasma corticosterone levels in the 4-week Alternating EE paradigm. Changes relative to the MIN group are highlighted. **B**, The EE variables examined in the present study included running, environmental complexity, social context (isolation and social enrichment) and stress-associated plasma corticosterone. **Voluntary running** strongly increased all stages of neurogenesis compared to exposure to a locked disc. Continuous voluntary running did not have a greater neurogenic effect than intermittent running. **Environmental complexity** (involving a combination of inanimate objects, social interactions and conformational novelty) did not affect basal or running-induced neurogenesis, but enhanced depolarization-associated c-fos expression within the granule cell layer (likely due to **Social interactions**) and decreased plasma corticosterone concentrations (likely due to physical **Complexity** associated with tunnels and toys). Chronic differences in the levels of **stress-associated corticosterone** had no detectable positive or negative effects on running-induced neurogenesis and were not responsible for preventing effects of environmental complexity on neurogenesis. The baseline environment, a Locked disc, had no detectable impact on any stage of neurogenesis, neuronal activation or corticosterone levels when compared to a completely impoverished environment. Thus, the Alternating EE paradigm enables dissociation of the impacts of distinct elements of environmental enrichment.

EE has gained widespread use as a means for enhancing brain function, both as an experimental tool and for rehabilitative therapy. However, a lack of understanding of this important phenomenon has led to the use of widely varying EE paradigms, with little comprehension of the potential consequences. It has therefore become imperative to better define the contributions of individual EE variables to specific underlying neural mechanisms [54], including adult neurogenesis. Here, we developed a novel “Alternating EE” paradigm that facilitates experimental isolation of key EE variables, and we used this paradigm to assess the impact of voluntary exercise, environmental complexity, stress and social interactions on hippocampal neurogenesis (summarized in Figure 7).

### Running

It is well established that exposure to an enriched environment containing a running wheel enhances adult hippocampal neurogenesis [31]–[33]. The picture that has emerged from such studies is that running itself is likely to be the prime positive regulator of neurogenesis within such an environment [31]–[33]. Unfortunately, extrapolating clear conclusions from data across previously published studies is hampered by differences among uncontrolled variables in the diverse EE paradigms that have been used. For instance: group housing is often considered a more naturalized environment, but it introduces significant social variables and prevents acquisition of individualized running data; few previous studies have used a locked running apparatus to control for possible running-independent influences; and conclusions regarding the effects on adult neurogenesis have often been based on only a single stage analysis of the neurogenic pathway (typically BrdU-NeuN to label the total number of newly generated neurons).

Our data confirm and extend upon previous conclusions concerning the role of running. As expected, findings obtained using our Alternating EE paradigm are consistent with previous studies showing that running is indeed the principle neurogenic stimulus within an enriched environment [31]–[33]. We were able to rule out any significant environmental complexity component of the running apparatus, as we controlled for the presence of the running disc itself. Our data revealed that the neurogenic effects of a 4-week running paradigm were not improved by a *Complex* environment, were not dependent on social context, and were not affected by stress-associated corticosterone levels (discussed individually below). Interestingly, we found that continuous voluntary running (7 d/week) did not elicit greater increases in neurogenesis than intermittent running (3 d/week), and that mice using running discs had comparable levels of neurogenesis as those using running wheels, despite having run 80% greater distances; both these data suggest a running-induced plateau in neurogenesis, a concept that has been previously suggested in a study by Naylor and colleagues [55]. Interestingly, at the end of our 4 week paradigm, the neurogenic effects of running on CD1 mice remained significant at all 4 stages of the neurogenic process (proliferation, neuroblasts, immature post-mitotic neurons, and cell survival); this contrasts previous findings using group-housed C57BL/6 mice, which exhibited a proliferative peak after 3–10 days that returned to baseline after 32 days [41].

Our results also showed that, despite significantly increasing expression of all markers of neurogenesis, 4 weeks of running unexpectedly decreased the total number of DG cells expressing c-fos (a surrogate marker of neuronal depolarization). We speculate that this reduction in depolarized neurons may be a by-product of the increased proliferation by transit-amplifying progenitors and neuroblasts, temporarily delaying the production of mature neurons. Consistent with this scenario, recent studies have shown that running increases immediate-early gene expression at longer 5–7 week timepoints [56], [57].

### Environmental complexity

Environmental complexity has been shown to positively affect a variety of neural parameters, including electrophysiological characteristics, depression, and learning and memory functions [54], [58]. However, mixed conclusions have been reached regarding whether a complex environment acts on adult neurogenesis, with some studies suggesting that it may have stage-specific effects on adult neurogenesis that are dissociable from the effects of exercise [34]–[36], [38], [59], [60] and other studies indicating that it does not appreciably modulate neurogenesis in comparison to running [31]–[33]. These conflicting views have likely arisen because environmental complexity has generally not been well isolated as a variable. Previous experimental designs could not exclude the possibility that observed effects of a complex environment were actually due to variables such as physical activity and social housing/isolation, or conversely, that a lack of observed effects was because basal neurogenesis levels had been raised by factors such as inanimate cage constituents, enriched feeding paradigms, handling or behavioral testing.

The *Complex* environment used in the present study was multi-factorial in nature, consisting of social housing (3 mice per cage), tunnels whose orientations were rotated 4 times per week, and a locked running apparatus (igloo and running disc). Thus, this environment possesses inanimate objects, social enrichment and conformational novelty. However, exposure to this environment did not affect any of the 4 stages of the neurogenic pathway examined. When combined with intermittent exposure to a running disc, it also did not potentiate running-induced neurogenesis. Importantly, control experiments allowed us to exclude a variety of factors that could possibly have masked the effects of the complex environment: by using CD1 mice that have low baseline neurogenesis, by testing for any effects of the locked disc environment itself, and by assessing possible neurogenic influences of daily handling or the use of vertical running wheels vs. horizontal running discs.

While the *Complex* environment did not alter any parameters of neurogenesis that we tested, two highly significant effects were detected. First, it increased depolarization-associated c-fos expression within the GCL by 2–3 fold. The upregulation of c-fos expression was detectable in both the inner and outer portions of the granule cell layer (generated during adulthood and early postnatally, respectively) and occurred to a similar extent in both the rostral (dorsal) and caudal (ventral) hippocampus. Since social housing by itself stimulated a similar increase in c-fos expression, social interactions are likely to be the c-fos-inducing component of the Complex environment. Second, the Complex environment strongly reduced levels of stress-associated plasma corticosterone. The suppression of plasma corticosterone occurred to a similar extent regardless of whether or not mice had access to a running disc. Since neither a locked disc nor social housing alone caused a reduction in corticosterone, it is likely that the tunnels and/or their regularly changing orientation is involved in mediating this effect.

We conclude from these data that environmental complexity as it is typically used in rodent models is not a significant or persistent regulator of hippocampal neurogenesis, and that its previously reported effects on neural function may instead be mediated by improved neuronal activation and/or by stress reduction. A possible caveat to its lack of neurogenic effects is that, despite being multi-factorial, the *Complex* environment might still be considered a mild or restricted form of cognitive stimulation. For example, previous work has indicated that learning-associated stimuli can enhance hippocampal neurogenesis via increased cell survival [61], [62]. In future experiments using this Alternating EE paradigm, it will be of interest to determine whether more focused or intensive types of cognitive stimulation can affect neurogenesis (i.e., maze training, controlled appropriately for exercise levels).

### Stress

Stress is generally associated with strong negative effects on neural functions, including learning and memory [10], [13], [63]. Activation of the hypothalamic-pituitary-adrenal (HPA) axis represents one of the key mechanisms underlying stress-associated effects, triggering release of adrenal-derived corticosterone into the circulation, from where it can enter the brain and activate the high levels of glucocorticoid and mineralocorticoid receptors found within the hippocampus [64]. Adult neurogenesis is among the hippocampal parameters regulated by stress-induced corticosterone. Considerable data support a negative correlation between corticosterone levels and dentate gyrus proliferation and neurogenesis [65]–[67], and stress-associated corticosterone can suppress running-induced neurogenesis in the short term [42]. However, the relationship between stress and adult neurogenesis is complex, as running is itself often considered a type of mild stressor, and has been associated with HPA activation and corticosterone release [42], [49]. Furthermore, adult neurogenesis appears to play a feedback role in regulating stress responses, as hippocampal neurogenesis has been implicated in the mood-stabilizing effects of antidepressant treatments [68] and inhibition of neurogenesis alters the responses to stress [69]–[71].

Plasma corticosterone levels were significantly altered across our experimental groups. Remarkably, neurogenesis markers (proliferation, neuroblast, immature neurons, survival) were comparably increased in all three running groups (I-RUN, C-RUN, MAX), despite the fact that the socially isolated I-RUN and C-RUN groups exhibited chronic 15-fold higher plasma corticosterone levels than the MAX group; thus, while an increase in corticosterone can negate running-induced neurogenesis in the short-term [42], it does not appear to have any significant long-term neurogenic impact. Exposure to an intermittent complex environment, either alone (I-CPX) or in combination with intermittent running (MAX), was sufficient to bring down plasma corticosterone, demonstrating that the lack of neurogenic effects of the complex environment observed in our study cannot be explained by high stress levels. While these observations may not apply to all types of stressors, the maintenance of high neurogenesis in the presence of chronically increased plasma corticosterone indicates that corticosterone-based mechanisms are unlikely to mediate long-term stressor effects on hippocampal neurogenesis.

### Social context

Group housing has long been used as a key component of EE [5], [31] and contributes to adaptive behavioral and anatomical changes in both the developing and adult brain [72], [73]. Conversely, social isolation has a significant negative impact on brain development and function [74]. In terms of hippocampal neurogenesis, many aspects of social context have been shown to affect neurogenesis, including social isolation [37], [42], psychosocial stress [50], [75]–[82], dominance hierarchy [83], social instability [84], reproductive behavior [85] and parenthood [86]–[89]. Of particular relevance here, previous work has shown that a social environment is capable of buffering stress-induced inhibition of neurogenesis, and at least in the short term, can be essential for allowing a running-induced up-regulation of adult neurogenesis [42].

The data obtained in the present study indicates that social context has limited long-term influence on running-induced neurogenesis. Social housing of mice in an empty cage (SOC mice) stimulated an increase in only NeuroD-expressing neuroblasts. Conversely, I-RUN and C-RUN mice displayed robust running-induced increases in neurogenesis, despite being completely socially isolated. Intermittent exposure to the social housing of the complex environment suppressed corticosterone levels in the isolated running group (MAX), supporting the idea that social housing can act to buffer the effects of stress; in spite of this, groups with intermittent social housing (i.e., I-CPX and MAX) did not exhibit increases in either basal (I-CPX) or running-induced (MAX) neurogenesis. Thus, social housing has limited pro-neurogenic activity, and social isolation is likely to only exert a temporary anti-neurogenic influence on running-induced neurogenesis [42].

### A novel Alternating EE paradigm for isolating individual EE variables

Few studies have attempted to dissect the impact of multiple EE variables within the same set of experiments. This is likely owing to the technical challenge associated with effectively isolating each variable, because “single” variables are typically multifactorial (a running wheel can serve as a source of both environmental complexity and physical activity, for example). We described here the development of a novel Alternating EE paradigm that enabled us to obtain a clearer idea of how each EE component impacts neurogenesis.

A major strength of this paradigm comes from its alternating nature. Mice are exposed to each environment for a pre-determined period of time (in the present case, alternating each day between one of two environments); consequently, statistical comparisons are made between groups that have been exposed to *identical conditions for about 50% of their time*. The intermittency of the approach also allows the possibility of a graded (intermittent vs. continuous) exposure to individual EE variables.

A range of additional technical considerations was also incorporated into the experimental design. While C57BL/6 mice have been the strain of choice in most mouse studies, a recent strain comparison revealed that C57BL/6 mice have the highest baseline neurogenesis level of 12 inbred strains examined, and thus exhibit the smallest EE-induced increase in adult neurogenesis [43]. To ensure that high baseline neurogenesis does not mask more subtle neurogenic effects of EE variables, we used male CD1 mice, a commonly used outbred mouse strain that we found exhibits low baseline neurogenesis and significant running-induced increases that are detectable even with low numbers of mice per group. Animals were individually housed during their exposure to running discs, ensuring that running data could be collected for each animal. The use of a locked disc group allowed controlling for the environmental complexity component of the running disc. Calretinin, a late marker that is expressed transiently in postmitotic, newly generated DG neurons [44], [45], as well as a more traditional BrdU-incorporation strategy, were used as independent measures of the total number of surviving newly born neurons. Finally, our control experiments revealed that inherent aspects of this Alternating EE paradigm, such as daily handling of the mice or the use of horizontal running discs versus vertical running wheels, had no detectable effects on the measured outcomes.

### Summary

The present study investigated the contributions of individual EE variables to adult hippocampal neurogenesis. A novel Alternating EE paradigm was developed that enabled us to effectively isolate single EE variables, that yielded consistent data, and whose design allows for wide flexibility in experimental parameters. Our data confirm and extend the understanding of the central role of voluntary running in the pro-neurogenic effects of EE. In contrast to some previous reports, we found that environmental complexity did not directly regulate or enhance running-induced neurogenesis; however, it did produce a significant stimulation of c-fos expression in the GCL, suggesting that it acts by enhancing the activity or integration of neurons into hippocampal circuitry. The complex environment also reduced plasma corticosterone concentrations, acting to buffer stress. Social context and chronically elevated circulating stress hormones (EE variables that can have significant effects on overall brain function) did not have long-term effects on basal or running-induced neurogenesis. The Alternating EE paradigm represents a useful experimental system for optimizing EE-based approaches in diverse contexts, such as developmental disorders, depression, aging, neurodegenerative diseases and rehabilitation following CNS lesions.
